# Discovery of Quercetin and Its Analogs as Potent OXA-48 Beta-Lactamase Inhibitors

**DOI:** 10.3389/fphar.2022.926104

**Published:** 2022-06-22

**Authors:** Yuejuan Zhang, Cheng Chen, Bin Cheng, Lei Gao, Chuan Qin, Lixia Zhang, Xu Zhang, Jun Wang, Yi Wan

**Affiliations:** ^1^ Microbiology Institute of Shaanxi, Xi’an, China; ^2^ Engineering Center of Qinling Mountains Natural Products, Shaanxi Academy of Sciences, Xi’an, China; ^3^ College of Forestry, Northwest A&F University, Yangling, China; ^4^ MOE Key Laboratory of Cell Activities and Stress Adaptations, School of Life Sciences, Lanzhou University, Lanzhou, China; ^5^ Clinical Laboratory, Shaanxi Provincial People’s Hospital, Xi’an, China

**Keywords:** antibiotic resistance, β-lactamase, OXA-48, inhibitor, flavonoid, quercetin

## Abstract

Carbapenem resistance in *Enterobacteriaceae* caused by OXA-48 β-lactamase is a growing global health threat and has rapidly spread in many regions of the world. Developing inhibitors is a promising way to overcome antibiotic resistance. However, there are few options for problematic OXA-48. Here we identified quercetin, fisetin, luteolin, 3′,4′,7-trihydroxyflavone, apigenin, kaempferol, and taxifolin as potent inhibitors of OXA-48 with IC_50_ values ranging from 0.47 to 4.54 μM. Notably, the structure-activity relationship revealed that the substitute hydroxyl groups in the A and B rings of quercetin and its structural analogs improved the inhibitory effect against OXA-48. Mechanism studies including enzymatic kinetic assay, isothermal titration calorimetry (ITC), and surface plasmon resonance (SPR) analysis demonstrated that quercetin reversibly inhibited OXA-48 through a noncompetitive mode. Molecular docking suggested that hydroxyl groups at the 3′, 4′ and 7 positions in flavonoids formed hydrogen-bonding interactions with the side chains of Thr209, Ala194, and Gln193 in OXA-48. Quercetin, fisetin, luteolin, and 3′,4′,7-trihydroxyflavone effectively restored the antibacterial efficacy of piperacillin or imipenem against *E. coli* producing OXA-48, resulting in 2–8-fold reduction in MIC. Moreover, quercetin combined with piperacillin showed antimicrobial efficacy in mice infection model. These studies provide potential lead compounds for the development of β-lactamase inhibitors and in combination with β-lactams to combat OXA-48 producing pathogen.

## Introduction

β-Lactams, including penicillins, cephalosporins, and carbapenems, are the most widely used antibiotics in clinics because of their high effectiveness and low toxicity (Zapun et al., 2008; Davies and Davies, 2010). However, the overuse of antibiotics has led to the emergence of multidrug-resistant (MDR) pathogens expressing β-lactamases that hydrolyze the pharmacophoric β-lactam ring of the antibiotics (Bush, 2010; Wright and Poinar, 2012). The β-lactamases are grouped into classes A-D based on their amino acid sequence homology and enzyme mechanisms. Class A, C, and D β-lactamases are serine β-lactamases (SβLs) that inactivate β-lactam antibiotics through a serine hydrolase mechanism, whereas class B β-lactamases are metallo-β-lactamases (MβLs) that utilize zinc ions for hydrolysis (Ambler, 1980; Bush and Jacoby, 2010). Currently, the combination of β-lactam antibiotics with β-lactamase inhibitors is a promising strategy to overcome antibiotic resistance.

OXA-48 is the main class D β-lactamase which was first isolated in Turkey in 2001 and has since been rapidly spread around the world (Poirel et al., 2004). It is frequently identified in *Escherichia coli, Enterobacter cloacae*, *Citrobacter freundii*, *Providencia rettgeri*, and *Klebsiella pneumoniae* (Nordmann and Poirel, 2014; Arana et al., 2015). OXA-48 has the ability to hydrolyze penicillins and carbapenems, but has weak or no activity against cephalosporins (Docquier et al., 2009). To date, none but avibactam is active against carbapenem-resistant *Enterobacteriaceae* producing OXA-48 in clinic (Liscio et al., 2015), whereas other commercial β-lactamase inhibitors, such as clavulanic acid, sulbactam, and tazobactam are chiefly active against class A and C SβLs (Drawz and Bonomo, 2010; Bush and Bradford, 2019). Unfortunately, resistance to avibactam has already been observed in clinic (Shields et al., 2017). Consequently, there is an urgent need to develop efficient inhibitors of OXA-48 to restore the efficacy of β-lactams.

**FIGURE 1:**
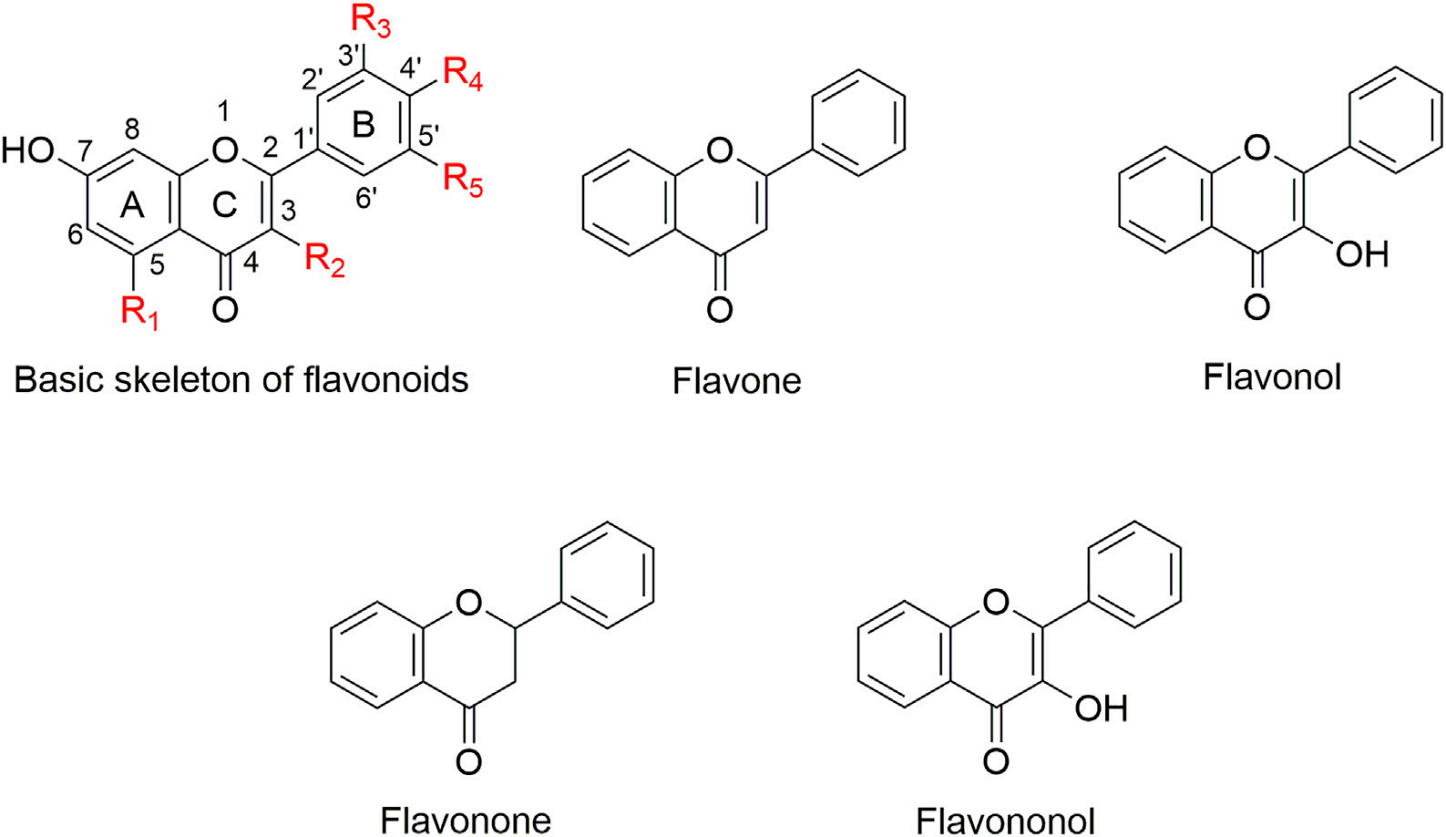
The basic skeleton of flavonoids and flavonoids used in this study.

Natural products have played an important role in the history of drug discovery (Garg et al., 2017). Among 1000s of natural bioactive compounds, flavonoids whose basic structure is a diphenylpropane (C6–C3–C6) skeleton (Farhadi et al., 2019), are a large group of structurally diverse natural products that occur widely in vascular plants (Schroeter et al., 2003; Ding et al., 2020) (Figure 1). Quercetin is a major flavonol compound with a variety of biological activities and important therapeutic applications (Chimenti et al., 2006; Lu et al., 2006; Fahlman and Krol, 2009). Recent studies have shown that quercetin restores antimicrobial susceptibility to meropenem among pathogenic *E. coli* and *Klebsiella pneumoniae* (Pal and Tripathi, 2020), and exhibits synergy with amoxicillin and ceftazidime against *Staphylococcus epidermidis* and *Streptococcus pyogenes*, respectively (Siriwong et al., 2015; Siriwong et al., 2016). In addition, quercetin (Riviere et al., 2020), baicalin (Shi et al., 2019), and isoliquiritin (Wang et al., 2020) confer efficacy in inhibiting MβL NDM-1. However, quercetin and its structural analogs have not yet been reported as potential inhibitors of class D carbapenemases.

In the present study, the preliminary cell-based screen was performed to search for inhibitors of OXA-48 from a natural product library. We successfully identified quercetin and its structural analogs as potent OXA-48 inhibitors. The inhibitory activities were tested against the purified OXA-48 enzyme. Representative quercetin was selected to investigate the inhibition mode by enzymatic kinetics, isothermal titration calorimetry (ITC), surface plasmon resonance (SPR), and molecular docking. The antibacterial activity of quercetin in combination with β-lactam was evaluated both *in vitro* and *in vivo*.

## Materials and Methods

### Bacterial Strains and Compound Preparation

The bacterial strains used in this study are listed in Table 1. The *bla*_oxa-48_ gene was synthesized by Sangon Biotech (Shanghai, China) and cloned into the pET26b plasmid for overexpression of OXA-48. *E. coli* BL21(DE3) cells were obtained from Novagen (Germany). *E. coli* BW25113 Δ*acrA* and Δ*bamB* were obtained from the *E. coli* Genetic Stock Center with stock codes 11843 and 9989, respectively. The piperacillin and imipenem used in this study were purchased from Sigma. All other chemicals were of analytical grade.

**TABLE 1:** Bacterial strains used in this study.

Strain name	Relevant genotype	Reference or source
BW25113	*E. coli* F- *Δ(araD-araB)567 ΔlacZ4787(::rrnB-3) λ-rph-1 Δ(rhaD-rhaB)568 hsdR514*	Keio Collection (Datsenko and Wanner, 2000)
BW25113 ∆*bamB*	BW25113 *bamB*::Kan	Keio Collection (Baba et al., 2006)
BW25113 ∆*acrA*	BW25113 *acrA*::Kan	Keio Collection (Baba et al., 2006)
BW25113 ∆*acrA ∆bamB*	BW25113 *bamB acrA*::Kan	This study
BL21 (DE3)	*E. coli* F- *ompT hsdS*_*B*_ (*r*_*B*_^*−*^ *m*_*B*_^*−*^) *gal dcm* (DE3)	Novagen

All compounds were purchased from commercial vendors. The HPLC chromatographs of flavonoids were displayed in Supplementary Figure S1. The stock solutions of the compounds were dissolved in dimethyl sulfoxide (DMSO) and diluted to various concentrations. The tolerance of OXA-48 to DMSO was tested, and less than 5% DMSO was used for all reactions.

### Development of an Efflux-Deficient Strain to Screen Inhibitors of OXA-48

The gene deletion within this study was performed as described previously (Datsenko and Wanner, 2000; Cox et al., 2017). *E. coli* BW25113 ∆*bamB* was transformed with the pCP20 plasmid to remove kanamycin cassette from the ∆*bamB* region. The ampicillin-resistant transformants were selected at 30°C, and further cultured at 42°C to remove all antibiotic resistance. The resulting strain was transformed with the pKD46 plasmid using the electroporation method. To remove the *acrA* gene, a region encompassing 500 bp upstream and downstream of ∆*acrA* was amplified and then transformed into the pKD46-harboring ∆*bamB* strain. Successful knockout transformants were selected, and the sequences were amplified by PCR and confirmed by DNA sequencing (Sangon, Shanghai, China). The kanamycin cassette from the *acrA* region was further removed by transformation with the pCP20 plasmid. The obtained strain was *E. coli* BW25113 *∆acrA∆bamB* mutant.

The *bla* promoter was amplified from pET22b, double-digested with the restriction enzymes *Xba* I and *Bgl* II and ligated to pET26b, resulting in the recombinant low-copy-number plasmid. The full-length *bla*_*oxa-48*_ with the native leader sequence was cloned into the obtained plasmid with the restriction enzymes *Nde* I and *Xho* I. The construct was verified by DNA sequencing. The resultant vector was then transformed into *E. coli* BW25113 *∆acrA∆bamB* to screen inhibitors of OXA-48. For the oligonucleotides used for gene deletion and plasmid construction, *see* Supplementary Table S1 in the supplemental material.

### Recombinant Protein Expression and Purification of OXA-48

The recombinant pET26b-OXA-48 plasmid was transformed into *E. coli* BL21(DE3). The OXA-48 protein was cultured in LB media with kanamycin (100 mg/L) for 3–4 h at 37°C and IPTG was added to induce expression at 20°C overnight. The cells were collected by centrifugation (14,000×*g*, 4°C, 30 min) and then sonicated. The lysate was loaded on a HiTrap SP HP column equilibrated with 20 mM HEPES (pH 7.0). The target protein was eluted with buffer containing NaCl at a concentration of 200 mM, and then concentrated by using an Amicon centrifugal filter. A size-exclusion chromatography using a HiLoad Superdex 75 column (GE Healthcare) was performed to further purify OXA-48 with a running buffer consisting of 20 mM HEPES (pH 7.0) supplemented with 200 mM NaCl. The concentration of purified OXA-48 was determined by UV spectrophotometry according to Lambert–Beer’s Law with an extinction coefficient of 65,430 M^−1^ cm^−1^ at 280 nm.

### Kinetic Experiments

The inhibitory activity of compounds against the purified OXA-48 was tested on a BioTek HTS reader. The reactions were conducted in 0.1 M phosphate buffer (pH 7.0) supplemented with 50 mM NaHCO_3_ at 25°C with a final assay volume of 100 μL. The concentrations of enzyme and substrate were 12 nM and 200 μM, respectively. The initial reaction rates in the absence and presence of the compound were monitored at 495 nm in triplicate. The inhibitor concentration causing a 50% decrease in enzyme activity (IC_50_) was determined by plotting the percent inhibition against the log_10_ of the inhibitor concentration and fitting the data in a dose–response-inhibition model using GraphPad Prism 7.0.

The inhibition mode of quercetin and the inhibition constant (*K*_i_) were determined using a Lineweaver–Burk plot by fitting the initial rate versus the substrate concentration at each inhibitor concentration using SigmaPlot 12.0. The nitrocefin concentrations were 25, 50, 75, and 100 μM, respectively. The initial velocity was determined by measuring the IC_50_ as described above. All reaction rates were determined in triplicate.

### Partition Ratio Measurements

OXA-48 β-Lactamase was incubated at a final concentration of 12 nM with serial dilutions of quercetin dissolved in 0.1 M phosphate buffer (pH 7.0), 50 mM NaHCO_3_ and 5% DMSO, respectively. After 1 h of incubation, the reaction was initiated in a 96-well plate by mixing 95 µL of preincubation mixture and 5 µL of 2 mM nitrocefin immediately. The absorbance of hydrolyzed nitrocefin was monitored at 495 nm at 1 s intervals for 10 min with a BioTek HTS reader in triplicate. Wells lacking β-lactamase were also used as blanks. The average progress curve was obtained by subtracting the baseline without enzyme. The initial rate of the progress curve was calculated according to the first 60 s. The partition ratio was defined as the ratio of quercetin to OXA-48 (*I*:*E*) necessary to inhibit the hydrolysis of nitrocefin by greater than 90%.

### Isothermal Titration Calorimetry Characterization

Isothermal titration calorimetry (ITC) measurements were performed as described previously (Zhang et al., 2018; Chen et al., 2022). OXA-48, imipenem, and quercetin were prepared in 30 mM Tris, 5 mM NaHCO_3_ (pH 7.5) supplemented with 5% DMSO. The final concentration of OXA-48 was 10 nM, that of imipenem was 1 mM, and that of the inhibitor varied from 0 to 40 µM. The solutions were degassed by centrifugation at a speed of 14,000 × *g* for 5 min before injection. The enzyme and inhibitor samples were preincubated for 1 h and then loaded into the sample cell. Imipenem (38 µL) in the syringe was titrated into the sample cell (210 µL) in a single injection mode. Heat flow (μcal/s) was recorded as a function of time. As a negative control, the reaction in the absence of quercetin was measured. The data were collected and analyzed with MicroCal Analysis Launcher Origin 7.0 software.

### Surface Plasmon Resonance Interaction Analysis

Surface plasmon resonance (SPR) experiments were performed on a Biacore T100 at 25°C. The running buffer consisted of 10 mM PBS (pH 7.3), 0.5% Tween-20, and 2.5% DMSO. OXA-48 solution was prepared in a concentration of 25 mg/mL in 10 mM PBS (pH 7.3) and immobilized on a CM5 chip using standard amine coupling to a level of approximately 5000 RU. Quercetin was dissolved in the running buffer and tested in 2-fold dilutions ranging from 12.5 to 200 µM with an injection time of 30 s and a dissociation time of 60 s. The data were analyzed using Biacore T100 Evaluation Software 2.0.

### Docking Studies

Molecular docking studies were performed using AutoDock 4.0 software, and the enzyme geometry in PDB code 4S2K was applied. Water molecules and ligands in the crystal structure were removed. The structures of the compounds were constructed by the ChemBio 3D program, and all possible torsion angles in the compounds were set free to carry out flexible docking. The compounds were docked into the crystal structures of OXA-48. The binding energy and interaction between each protein and compound including those for hydrophobic contacts and hydrogen bonds were found through molecular docking. The structures of the complexes were visualized and analyzed using PyMOL software.

### Susceptibility Testing of Antibiotics and Inhibitors

The recombinant plasmid containing the P_bla_ promoter and OXA-48 constructed above was then transformed into BW25113, BW25113 ∆*acrA*, BW25113 ∆*bamB*, and BW25113 ∆*acrA*∆*bamB*. The above strains were used to study the minimum inhibitory concentration (MIC). Antibiotic susceptibility tests were performed by the microdilution method following Clinical and Laboratory Standards Institute (CLSI) criteria (CLSI, 2015). Four *E. coli* strains expressing OXA-48 were used to assess the inhibitors. The susceptibility to piperacillin/imipenem combined with the compound at a concentration of 16 or 64 μg/mL was determined. The MIC is defined as the lowest drug concentration that completely inhibited bacterial growth after incubating the plates at 37°C for at least 16 h (Kang et al., 2020). Each measurement was conducted in triplicate. The fractional inhibitory concentration index (FICI) was determined according to the following formula: FICI = MIC (QT) in combination/MIC (QT) alone + MIC (PIP) in combination/MIC (PIP) alone. The drug interactions are defined as synergistic if the FICI is ≤0.5, indifferent if the FICI is >0.5 and ≤4.0, and antagonistic if the FICI is >4.0.

### Time Kill Assay

OXA-48-positive BW25113 ∆*acrA* ∆*bamB* (OXA-48) was cultured in LB broth to the logarithmic growth phase at 37°C. Each culture tubes were inoculated with 1 × 10^6^ CFU/mL and treated with PBS (control), piperacillin (64 μg/mL), or piperacillin plus quercetin (64 + 64 μg/mL), respectively. The bacteria were incubated at 37°C. At 0, 4, 8, 16, and 24 h, 100 μL of bacterial suspension were 10-folds serial diluted and plated onto LB agar plates to calculate colonies. All experiments were set for three parallels for each group.

### *In Vivo* Evaluation of Antibacterial Activity Using a Mouse Systemic Infection Model

The mouse systemic infection model was developed as previously reported (King et al., 2014; Chen et al., 2022). *E. coli* BW25113 ∆*acrA*∆*bamB* (OXA-48) were cultured to an OD_600_ value of 1.0 and then washed with PBS three times. A 150 μL of bacterial suspension was injected into the Kunming mice intraperitoneally at a dose of 1 × 10^9^ CFUs. After 1 h of infection, mice were divided into four groups randomly and treated with PBS+ 5% DMSO, piperacillin (10 mg/kg), quercetin (10 mg/kg), or a combination of piperacillin and quercetin (10 mg/10 mg/kg) by intravenous injection (*n* = 6 per group). To evaluate the bacterial load burden, mice were sacrificed, spleen and liver were collected after 24 h post-infection. The organs were homogenized in ice-cold PBS and cultured on LB agar to count the isolated colonies.

## Results

### Screening Assay for Natural Products That Inhibit the Activity of OXA-48

**FIGURE 2:**
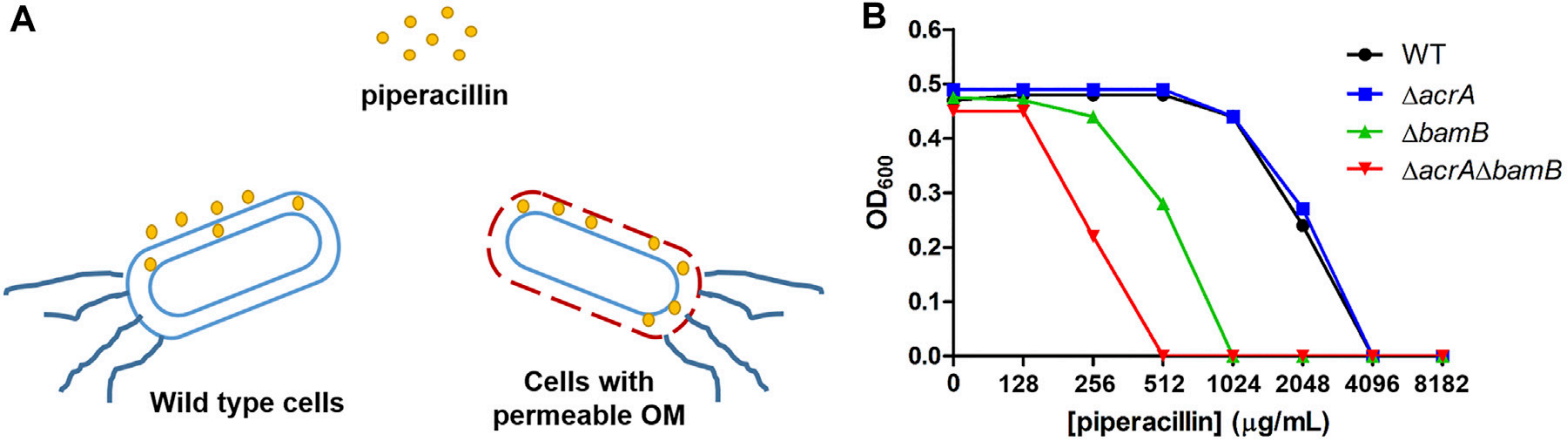
**(A)** Deletion of resistance genes increased the permeability of the outer membrane of *E. coli*. **(B)** MIC determination of piperacillin for wild-type (WT) *E. coli* and gene deletion mutants expressing OXA-48.

Efflux pumps and membrane proteins play an important role in the uptake of drug molecules in bacteria. Previous studies have shown that the disruption of *bamB* caused bacterial outer membrane biogenesis defects and facilitated the permeation of small molecules into bacteria (Ricci and Silhavy, 2012) and that *acrA* encodes the periplasmic adaptor protein in the AcrAB-TolC efflux pump system(Blair and Piddock, 2009). To establish a highly sensitive cell-based platform for screening inhibitors of carbapenemase OXA-48, we deleted the *bamB* and/or *acrA* genes in *E. coli* BW25113 by using the bacteriophage λ Red recombinase system (Datsenko and Wanner, 2000; King et al., 2014) (Figure 2A). The *bla*_OXA-48_ gene was cloned into the modified pET26b plasmid under the control of the P_bla_ promoter and then transformed into *E. coli* BW25113 deletion mutants*.* To test antibiotic susceptibility of the gene deletion strains, the MICs of wild type strain and these mutants (BW25113, BW25113 ∆*acrA,* BW25113 ∆*bamB,* and BW25113 ∆*acrA*∆*bamB* expressing OXA-48) against piperacillin were measured. We found that deletion of *bamB* alone facilitated the permeation of piperacillin, resulting in a 4-fold increase in the piperacillin susceptibility, while codeletion of *bamB* and *acrA* resulted in an 8-fold increase in the piperacillin susceptibility (Figure 2B).

The ∆*acrA*∆*bamB* (OXA-48) mutant strain was then used to screen for inhibitors of OXA-48. We monitored the growth curves of the test strain incubated with 0, 1/16×, 1/8×, 1/4×, 1/2×, and 1× MIC piperacillin. As shown in Supplementary Figure S2, 1/4× MIC (128 μg/mL) piperacillin was chosen for the screening assay in this study according to the minimal effective concentration principle. Approximately 150 plant natural products with a concentration of 40 μg/mL were screened in the presence of 128 μg/mL piperacillin. Around 15% of these molecules which exhibited antimicrobial activity in the absence of piperacillin were not considered further. Notably, quercetin restored antibiotic activity against the screening strain in combination with piperacillin but itself without bactericidal effect, indicating that quercetin may be an inhibitor of OXA-48.

### Evaluation of the Inhibitory Activity of Quercetin and its Structural Analogs Against OXA-48 *In Vitro*

**FIGURE 3:**
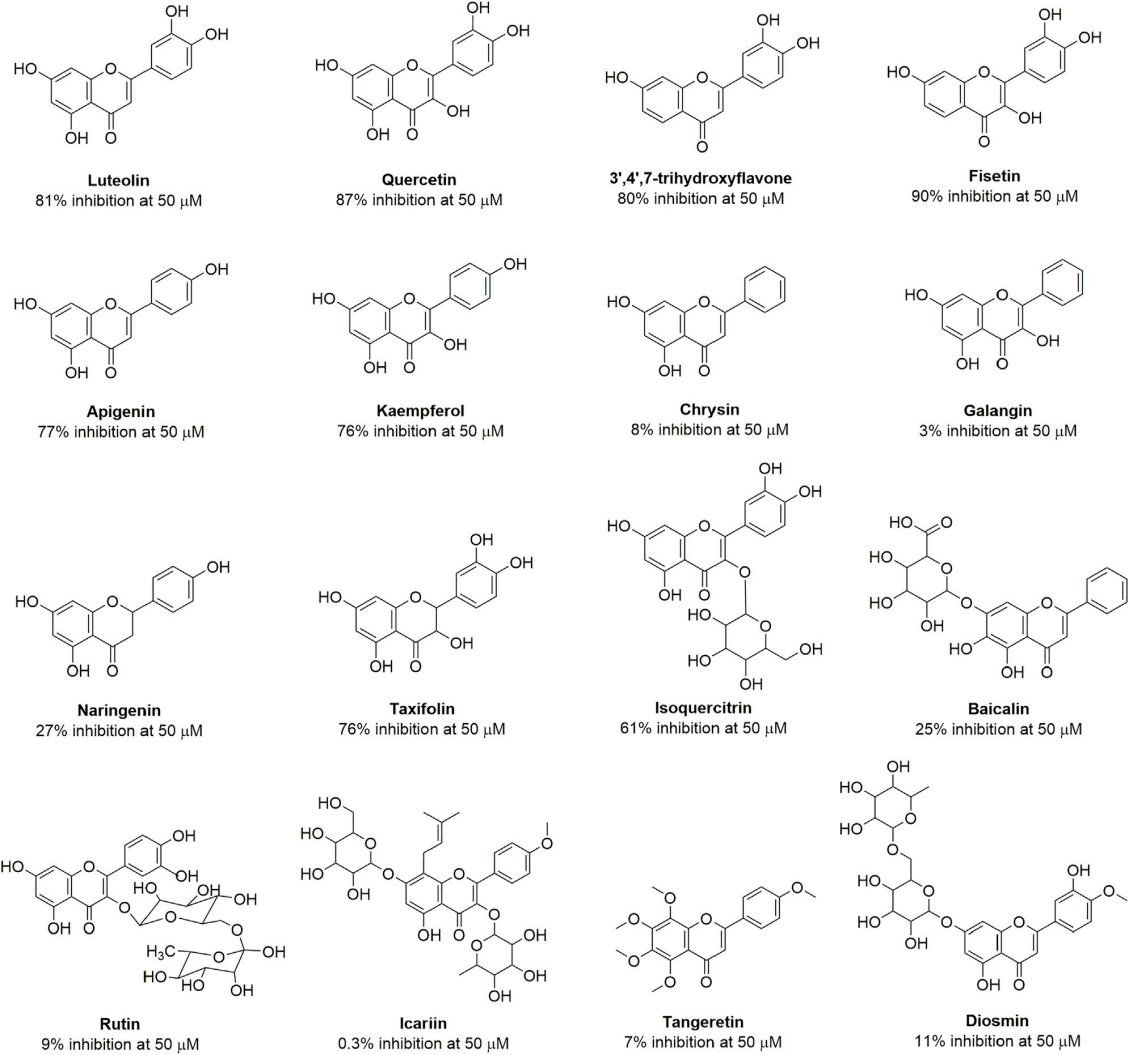
Structures of the flavonoids and their percent inhibition against OXA-48. Nitrocefin was used as the substrate.

Quercetin is a member of the flavonoid class with diverse structures obtained from nature, some of which have been used for the treatment of infectious diseases in traditional medical systems (Farhadi et al., 2019). To investigate whether quercetin and other flavonoids are OXA-48 inhibitors, the enzyme inhibition assays were conducted on a BioTek HTS reader using nitrocefin as a substrate. The concentrations of enzyme and substrate were 12 nM and 200 μM, respectively. The initial hydrolysis rate of nitrocefin was monitored at 495 nm. The percentage inhibition is defined as the enzyme activity in the absence of inhibitor (100%) minus the remaining activity in the presence of inhibitor. The structures of tested compounds and percent inhibition gained were shown in Figure 3. It can be seen that apigenin, luteolin, kaempferol, quercetin, taxifolin, fisetin, 3′,4′,7-trihydroxyflavone and isoquercitrin exhibited more than 50% inhibition of OXA-48 at a concentration of 50 μM. However, chrysin, galangin, rutin, diosmin, icarlin showed less than 20% inhibition.

Eleven of these compounds were selected to determine the IC_50_ against OXA-48. The data were presented in Table 2 and the profiles were shown in Supplementary Figure S3, which is in consistent with their percent inhibition. It is observed that quercetin, fisetin, luteolin, 3′,4′,7-trihydroxyflavone, apigenin, kaempferol and taxifolin showed excellent inhibitory effect against OXA-48 with IC_50_ values ranging from 0.47 to 4.54 μM. Among them, quercetin exhibited a dose-dependent inhibition of OXA-48 with an IC_50_ value of 1.47 µM, indicating that the capability of quercetin to restore the antimicrobial activity of piperacillin was due to its inhibition of OXA-48 activity.

**TABLE 2:** Inhibitory activity (IC_50_, μM) of flavonoids against OXA-48.

Compounds	Name	IC_50_ (µM)	Compounds	Name	IC_50_ (µM)
1	Fisetin	0.47 ± 0.03	7	Isoquercitrin	11.55 ± 1.22
2	Luteolin	0.55 ± 0.02	8	Chrysin	108 ± 11
3	Quercetin	1.47 ± 0.14	9	Galangin	216 ± 15
4	3′,4′,7-trihydroxyflavone	1.89 ± 0.11	10	Taxifolin	3.22 ± 0.23
5	Apigenin	3.64 ± 0.24	11	Naringenin	68.10 ± 4.12
6	Kaempferol	4.54 ± 0.05	—	—	—

### Structure-Activity Relationship

From the obtained IC_50_ values in Table 2, we determined the structure–activity relationships. The hydroxyl group in the B ring was found to be crucial to inhibitory activity. For example, chrysin (IC_50_ = 108 μM) and galangin (IC_50_ = 216 μM), which lack hydroxyl group in the B ring, exhibited much lower activity than apigenin (IC_50_ = 3.64 μM) and kaempferol (IC_50_ = 4.54 μM). The compounds with two hydroxyl groups showed better activity than those with a single hydroxyl group in the B ring. For instance, luteolin (IC_50_ = 0.55 μM), quercetin (IC_50_ = 1.47 μM) and fisetin (IC_50_ = 0.47 μM), which had hydroxyl groups disubstituted at C3′ and C4′, were more potent than apigenin (IC_50_ = 3.64 μM) and kaempferol (IC_50_ = 4.54 μM), which had a hydroxyl group substituted only at C4′. Moreover, flavonoids with a hydroxyl group substituted at R1 and R2 exhibited little effect on inhibitory activity. The activity levels of apigenin (IC_50_ = 3.64 μM) and quercetin (IC_50_ = 1.47 μM) were comparable to those of kaempferol (IC_50_ = 4.54 μM) and fisetin (IC_50_ = 0.47 μM). It is also suggested that the hydroxyl group at C3 or C5 is not essential for the inhibitory activity but one hydroxyl group at the positions may increase the compound’s biological action, such as fisetin (IC_50_ = 0.47 μM) and luteolin (IC_50_ = 0.55 μM) which exhibited higher inhibitory activity compared to quercetin (IC_50_ = 1.47 μM) and 3′,4′,7-trihydroxyflavone (IC_50_ = 1.89 μM). In addition, for saturated taxifolin (IC_50_ = 3.22 μM) and naringenin (IC_50_ = 68.1 μM), the absence of the double bond in the C ring resulted in lower activity than that of unsaturated quercetin (IC_50_ = 1.47 μM) and apigenin (IC_50_ = 3.64 μM). This may have been due to the 2,3-double bond in conjugation with the 4-keto group ensuring π-electron delocalization between the B- and C-rings (Leopoldini et al., 2004; Marsal et al., 2006; Topal et al., 2016). However, larger groups at position 3, such as in isoquercitrin (IC_50_ = 11.55 μM) and rutin (no inhibitory activity), led to weak activity against the enzyme, probably because of the steric hindrance generated by the replacement of the hydroxyl group. Collectively, the results indicated that the hydroxylation group in the C6-C3-C6 skeleton (the core structure of flavonoids) played a crucial role in the inhibition of OXA-48 and that the presence of the hydroxyl group in the A and B rings increased the inhibitory activity.

### Inhibitory Mechanism of Quercetin Against OXA-48

**FIGURE 4:**
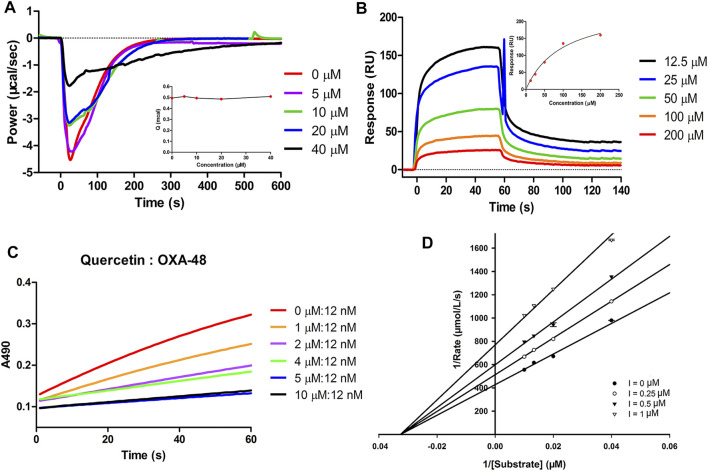
**(A)** Heat flow curves of imipenem hydrolysis by OXA-48 in the presence of quercetin at concentrations ranging from 0 to 40 μM using a single injection mode at 25°C monitored by ITC. The inset shows the total heat in the presence of quercetin at different concentrations. **(B)** Sensorgrams for the interaction of quercetin with OXA-48. The inset shows the SPR binding kinetics analysis of quercetin. **(C)** Progress curves for partition ratio measurement. OXA-48 at 12 nM was incubated with various concentrations of quercetin. **(D)** Lineweaver–Burk plots of OXA-48 catalyzed hydrolysis of the substrate in the absence and presence of quercetin at various concentrations.

Many studies have shown that quercetin not only has numerous pharmacological activities, such as anti-cancer, anti-viral, antioxidant, anti-inflammatory and anti-senescence effects (Patel et al., 2018; Shiva et al., 2018; Wang et al., 2021), but also is readily available and has low cytotoxicity (Yang et al., 2020). Meanwhile, in this study, quercetin in combination with MIC of piperacillin exhibited antibacterial activity against the OXA-48 positive strain, quercetin was therefore selected as a representative flavonoid to elucidate the possible inhibition mode against OXA-48. Isothermal titration calorimetry (ITC) was performed to assess the thermodynamic inhibitory effect of quercetin against OXA-48 (Zhang et al., 2018). Considering the catalytic efficiency values of imipenem (*k*_cat_/*K*_m_ = 624 ± 148 s^−1^mM^−1^) and nitrocefin (substrate for enzyme kinetic analysis) (*k*_cat_/*K*_m_ = 365 ± 71 s^−1^mM^−1^) for OXA-48 were comparable (Frohlich et al., 2019), imipenem was selected as the hydrolytic substrate for the ITC experiment. Different concentrations of quercetin were incubated with purified OXA-48 for 1 h in 30 mM Tris, 5 mM NaHCO_3_ (pH 7.5). The mixtures were loaded into the sample cell (210 µL). A 38 µL of the substrate (imipenem) in the syringe was titrated into the sample cell in a single injection mode. The heat flow values (μcal/s) of imipenem hydrolysis catalyzed by OXA-48 in the presence of different concentrations of inhibitor are shown in Figure 4A. The results revealed that the negative thermal power (*P*) appeared after the imipenem injection, indicating that the hydrolysis reaction was exothermic and that the hydrolysis of imipenem was inhibited by adding quercetin into the reaction mixtures. The total heat did not change with increasing inhibitor concentrations (Figure 4A inset), suggesting that quercetin reversibly inhibited OXA-48.

A surface plasmon resonance (SPR) assay was then performed to investigate whether quercetin inhibited the activity of OXA-48 by directly binding to OXA-48. OXA-48 enzyme was immobilized on a CM5 sensor chip with an immobilization level of approximately 5000 RU. Quercetin was injected at different concentrations (12.5, 25, 50, 100 and 200 µM) over the protein surface. As shown in Figure 4B, SPR characterization displayed an increase in the SPR signal (RU) with the increasing concentration of quercetin, revealing a dose-dependent interaction between quercetin and OXA-48. Based on these data, quercetin exhibited molecular interaction with OXA-48.

The partition ratio is the molar ratio of an inhibitor to a target enzyme required to achieve greater than 90% inhibition in sufficient time (Vallejo et al., 2016; Shapiro and Gao, 2021). To determine the partition ratio of quercetin, OXA-48 was incubated with increasing concentrations of quercetin and then the hydrolysis curves were monitored. Our results illustrated that the partition ratio value of quercetin with OXA-48 was approximately 420 at a 5 μM concentration of quercetin (Figure 4C). Some β-lactamase inhibitors with partition ratio over than 1 may be hydrolyzed by β-lactamase (Fisher et al., 1978). For instance, clavulanic acid and sulbactam have partition ratio values of 2,500 and 1,000 for KPC-2, respectively (Papp-Wallace et al., 2010). These results likely demonstrated that quercetin was to some extent hydrolyzed by OXA-48.

To determine the inhibition mode of the flavonoids, the inhibition kinetics of quercetin, fisetin and luteolin against OXA-48 in the presence of different concentrations of substrate were determined, and the inhibition modes were determined by generating Lineweaver-Burk plots. The Lineweaver-Burk plots of OXA-48-catalyzed hydrolysis of nitrocefin in the absence and presence of the compounds are shown in Figure 4D and Supplementary Figure S4. With the increase of compound concentration, the affinity (*K*_m_) did not change, but the maximum rate (*V*_max_) decreased. The results illustrated that quercetin, fisetin and luteolin noncompetitively inhibited the hydrolytic activity of OXA-48 with a *K*i value of 1.31 ± 0.21, 0.62 ± 0.06 and 0.82 ± 0.05 μM, respectively, which were consistent with the IC_50_ values.

### Docking Studies

**FIGURE 5:**
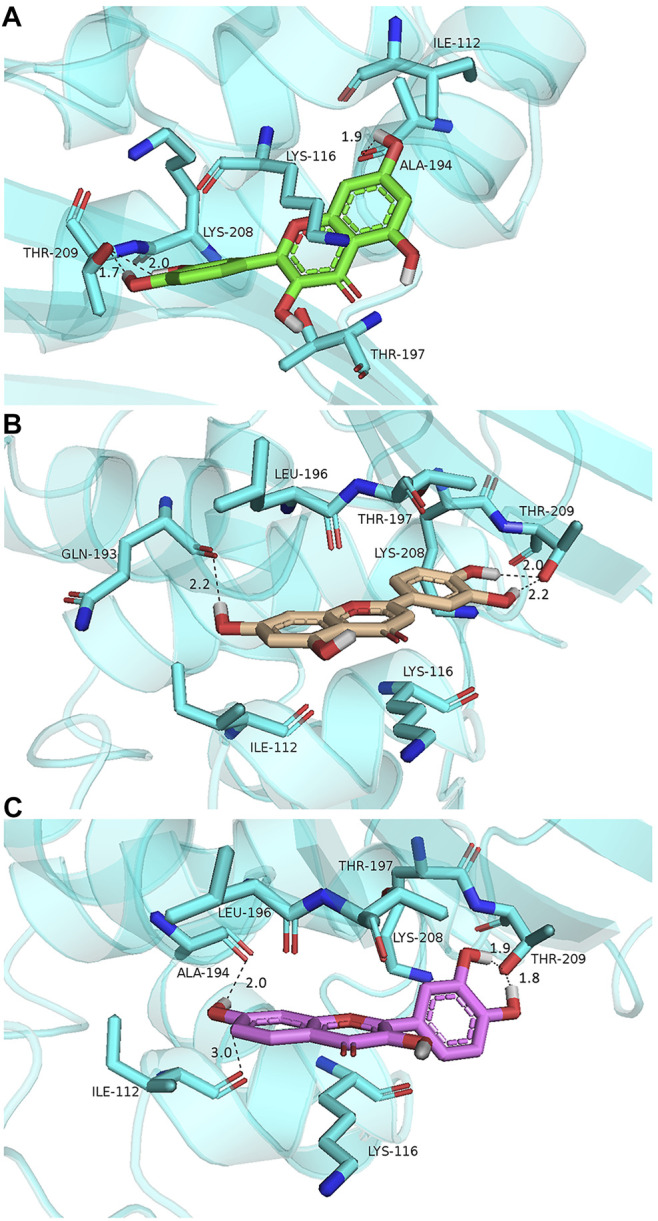
Low-energy docking conformations of quercetin (**A**, green), luteolin (**B**, gold), and fisetin (**C**, purple) docked into OXA-48 (PDB code 4S2K). The enzyme backbone is shown as a cartoon in cyan and relevant side chain residues are labeled and shown as sticks colored according to the atom (C, cyan; N, blue; O, red). The hydrogen-bonding interactions are indicated by dashed black lines. The figures were created using the PyMOL Molecular Graphics System.

To explore the potential binding modes of quercetin and its structural analogs as potent OXA-48 inhibitors, molecular docking was performed to analyze the modes by which quercetin, luteolin, and fisetin bound with OXA-48 (PDB code 4S2K). The conformations generated with PyMOL are illustrated in Figure 5, and the binding energies were −6.28, −6.21, and −7.14 kcal/mol for quercetin/OXA-48, luteolin/OXA-48, and fisetin/OXA-48 complexes, respectively. The three compounds bound to OXA-48 in the same overall orientation, interacting with residues from the β-sheet domain of the enzyme. As shown in Figure 5, in all complexes, the hydroxyl groups in the B ring of the compounds formed two hydrogen bonds with the side chain of Thr209 from strand β5, a residue highly conserved in class C and class D enzymes (Toth et al., 2016). Moreover, the 7-hydroxyl group in the A ring was found to have hydrogen-bonding interaction with Ala194 (Figures 5A,B) or Gln193 (Figure 5C) with a distance of 1.9, 2.2, and 2.0 Å, respectively. Furthermore, the 7-hydroxyl group in the A ring formed a hydrogen bond with Ile112 in the fisetin/OXA-48 complex with a distance of 3.0 Å (Figure 5C), contributing to a lower IC_50_ of fisetin against OXA-48. Apart from the hydrogen bonds, quercetin, fisetin and luteolin also formed hydrophobic interactions with the residues of Lys116 and Ile112 by hydrophobic interaction forces. Consequently, these docking studies demonstrated that the hydroxyl groups at the 3′, 4′ and 7 positions played a significant role in the effective inhibition, consistent with the findings that quercetin and luteolin as OXA-48 inhibitors exhibiting lower IC_50_ values than chrysin and galangin. These results provided useful insights into further optimization of the lead compounds of OXA-48.

### Antimicrobial Susceptibility Assays

To investigate whether the compounds could restore the antimicrobial activity of β-lactam, the MICs of β-lactam antibiotics against resistant Gram-negative bacteria in the absence and presence of compounds (fisetin, 3′,4′,7-trihydroxyflavone, luteolin or quercetin) were determined by using published guidelines described by CLSI (CLSI, 2015). Specifically, we used OXA-48 producing strains *E. coli* BW25113, BW25113 ∆*acrA*, BW25113 ∆*bamB*, and BW25113 ∆*acrA*∆*bamB* as resistant Gram-negative bacteria. Piperacillin, a member of the penicillin class, was used at increasing concentrations from 32 μg/mL to 4096 μg/mL. Imipenem, a carbapenem antibiotic, was applied at increasing concentrations ranging from 0.03 μg/mL to 4 μg/mL. The compounds were tested at 64 and 16 μg/mL.

The MIC results are presented in Table 3. All the tested compounds combined with β-lactam antibiotics displayed antimicrobial activity against BW25113 ∆*acrA*∆*bamB* (OXA-48) at the concentration of 64 μg/mL. Quercetin and 3′,4′,7-trihydroxyflavone showed an 8-fold reduction in the piperacillin MIC, while the MIC of piperacillin was decreased 2- and 4-fold in the presence of fisetin and luteolin, respectively. In contrast, quercetin, fisetin, and luteolin resulted in a 2-fold MIC decrease for imipenem at a dose of 64 μg/mL. For the ∆*bamB* (OXA-48) strain, 64 μg/mL compounds decreased the MIC of imipenem by 2-fold but did not affect the MIC of penicillin. However, none of the four compounds were able to reduce the MIC in BW25113 (OXA-48).

**TABLE 3:** Antibiotic MIC values for bacterial strains expressing OXA-48.

β-Lactam + inhibitor	*E.coli* BW25113 (OXA-48)		*E.coli* BW25113 ∆*acrA* (OXA-48)		*E.coli* BW25113 ∆*bamB* (OXA-48)		*E.coli* BW25113 *∆acrA∆bamB* (OXA-48)	
	16 mg/L	64 mg/L	16 mg/L	64 mg/L	16 mg/L	64 mg/L	16 mg/L	64 mg/L
Imipenem	4	4	4	4	4	4	4	4
Imipenem + FIS	4	4	4	2	4	2	2	2
Imipenem + TFA	4	4	2	1	2	2	2	1
Imipenem + LUT	4	4	4	2	4	2	4	2
Imipenem + QT	4	4	4	2	4	2	4	2
Piperacillin	4096	4096	4096	4096	1024	1024	512	512
Piperacillin + FIS	4096	4096	4096	4096	1024	1024	512	256
Piperacillin + TFA	4096	4096	2048	256	1024	1024	256	64
Piperacillin + LUT	4096	4096	4096	4096	512	512	512	128
Piperacillin + QT	4096	4096	2048	1024	1024	1024	256	64

IMP, imipenem; PIP, piperacillin; FIS, fisetin; TFA, 3′,4′,7-trihydroxyflavone; LUT, luteolin; QT, quercetin. The data are presented as the means form three independent experiments.

**FIGURE 6:**
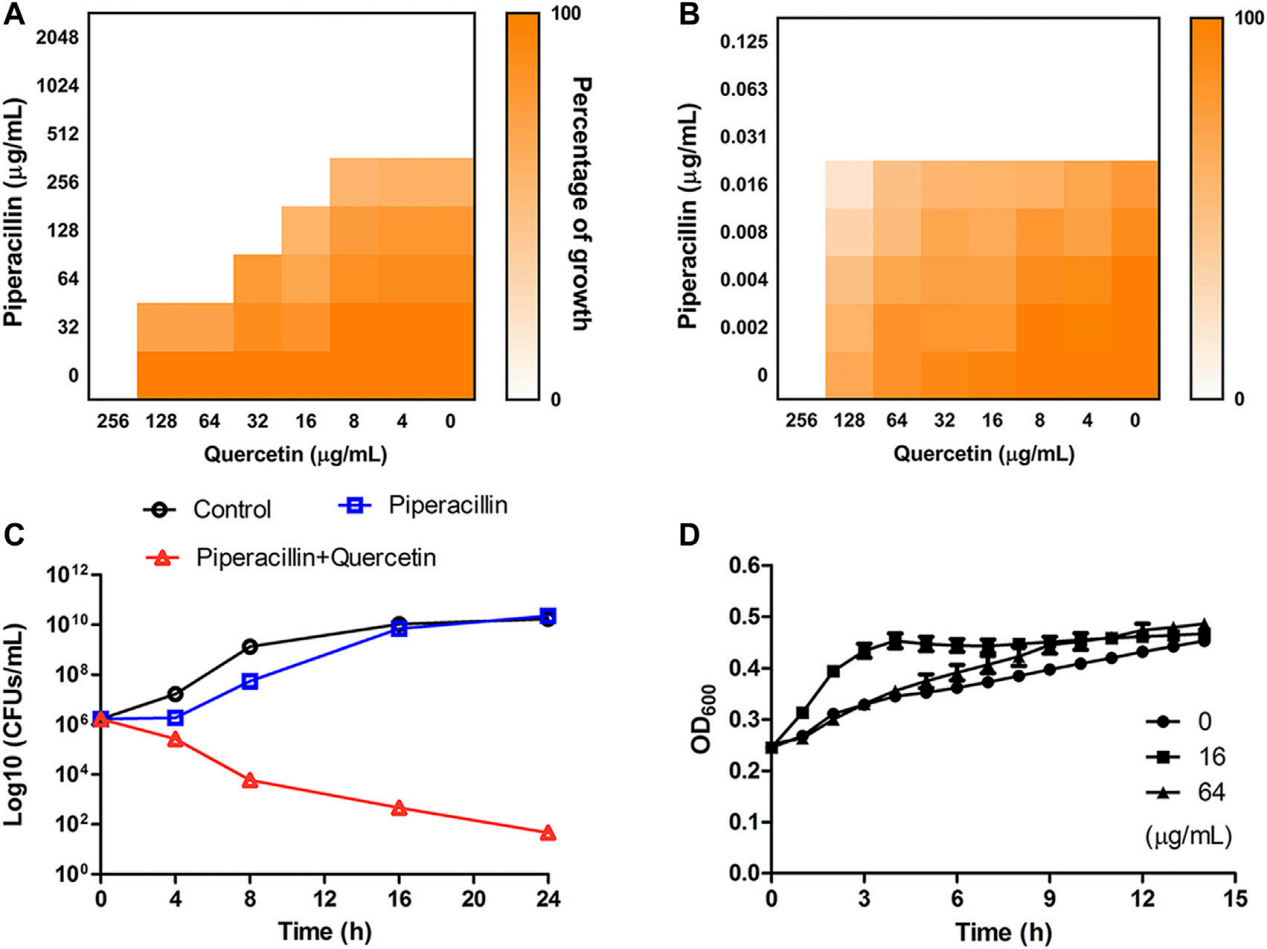
**(A**,**B)** Heatmap showing the growth inhibition of OXA-48-positive strain *E. coli* BW25113 ∆*acrA*∆*bamB* (OXA-48) **(A)** and OXA-48-negative strain ∆*acrA*∆*bamB* **(B)** in the presence of quercetin and piperacillin. **(C)** Time-kill curves for combination therapy of piperacillin and quercetin against *E. coli* BW25113 ∆*acrA*∆*bamB* (OXA-48). The concentrations of piperacillin and quercetin used in the assay were 64 and 64 μg/mL , respectively. **(D)** The growth curve of *E. coli* BW25113 ∆*acrA*∆*bamB* (OXA-48) in the absence and presence of quercetin.

We next performed checkerboard microdilution assays against OXA-48-positive strain BW25113 *∆acrA∆bamB* (OXA-48) and OXA-48-negative strain ∆*acrA*∆*bamB* to assess the synergistic antimicrobial effect. The fractional inhibitory concentration index (FICI) calculated for the quercetin-piperacillin combination showed a synergistic effect of the interactions between the two compounds against OXA-48-positive bacteria with a FICI value of 0.375 (Figure 6A). As expected, no synergy was observed in the sensitive strain (Figure 6B, FICI = 2.0) (FICI ≤0.5 is defined as synergistic effect). The synergy effect was further verified by time-kill curves. The bacterial colonies significantly decreased after exposure to the combination therapy of 64 μg/mL piperacillin and 64 μg/mL quercetin for 24 h (Figure 6C). To further investigate whether quercetin exhibits bactericidal ability against the tested bacteria, quercetin was added to the bacteria, and the growth curves were monitored. As shown in Figure 6D, quercetin at a concentration of 16 or 64 μg/mL did not inhibit the growth of the cells. These data revealed that quercetin in combination with piperacillin displayed a significant synergistic antimicrobial effect *in vitro*.

### *In Vivo* Antimicrobial Efficacy of Combination Therapy

**FIGURE 7:**
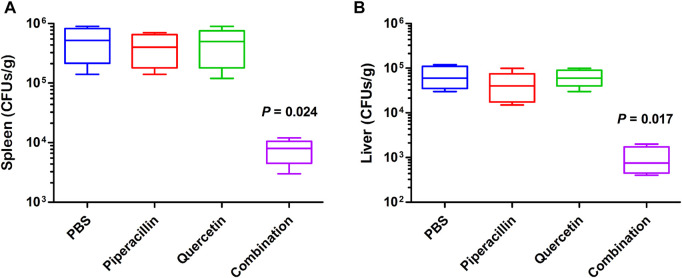
Antimicrobial efficacy of quercetin in combination with piperacillin *in vivo*. Monotherapy of PBS (control), piperacillin (10 mg/kg), quercetin (10 mg/kg) or combination therapy of piperacillin and quercetin (10 + 10 mg/kg) were treated to mice groups infected with *E. coli*-OXA-48, respectively. After 24 h of infection, the bacterial burden in the spleen **(A)** and liver **(B)** of mice were calculated by colony counting.

A mouse systemic *E. coli* infection model was employed to evaluate whether quercetin could restore the antimicrobial efficacy of piperacillin *in vivo*. Kunming mice were infected intraperitoneally by *E. coli* producing OXA-48 with a nonlethal dose. Monotherapy with piperacillin (10 mg/kg) and quercetin (10 mg/kg) and combination therapy (10 + 10 mg/kg) were performed on infected mice after 1 h post infection. After 23 h of post treatment, the bacterial burden in the spleen and liver of mice were determined, respectively. As shown in Figure 7, infected mice treated with piperacillin or quercetin monotherapy did not show a significant reduction of bacterial load compared with the untreated group. Notably, the combination treatment with piperacillin and quercetin decreased the bacterial burden in the liver and spleen (*p* < 0.05), demonstrating that combination therapy with piperacillin and quercetin has excellent efficacy as a therapeutic agent to treat OXA-48 producing pathogen.

## Discussion

Carbapenem-hydrolysing OXA-48 β-lactamase becomes one of the most severe threat of antibiotic resistance and compromises effective anti-infection treatment (Lee et al., 2016). Through relentless efforts, different OXA-48 inhibitors with many mechanisms of action have been reported. LN-1-255, a 6-alkylidene-2′-substituted penicillanic acid sulfone, was reported to inhibit OXA-48 and significantly reduce MICs of carbapenem (Vallejo et al., 2016). The 3-substituted benzoic acid derivatives were discovered to inhibit OXA-48 by bounding to Arg250, Arg214 and Tyr211 in the active site non-covalently (Akhter et al., 2018). Biphenyl-, naphthalene-, fluorene-, anthraquinone-, and azobenzene-based compounds were identified as OXA-48 non-covalent inhibitors with *K*i values below 50 μM according to an FBDD approach (Taylor et al., 2021). Garofalo et al. screened 4-ideneamino-4H-1,2,4-triazole and pyrazolo[3,4b]pyridine as competitive OXA-48 inhibitors with a reversible mechanism of action (Garofalo et al., 2021). Despite the advances in the development of OXA-48 inhibitors, there are limited options for OXA-48 in clinical application. Hence, developing OXA-48 inhibitors to restore the activity of β-lactam antibiotics is greatly needed.

Here we identified quercetin and its analogs inhibited the activity of OXA-48 effectively. Compared with previously reported inhibitors, quercetin and its analogs are inexpensive, easy to prepare and can be extracted and purified from plants in large quantities. Moreover, quercetin, fisetin and luteolin with potent inhibitory activity against OXA-48 exhibit no toxicity to the cells even at a concentration of 100 μg/mL quercetin (Hatahet et al., 2018) or 50 µM fisetin/luteolin (Hytti et al., 2015). Animal studies illustrated that quercetin and luteolin had little toxicity in mice with an LD_50_ value of 160 mg/kg (Sullivan et al., 1951; Taepongsorat et al., 2008) and 150 mg/kg (Ardeshir et al., 2018), respectively. Fisetin showed no toxicity at doses up to 2 g/kg in mouse acute toxicity assay (Currais et al., 2013). Therefore, quercetin and its analogs may be potential safe inhibitors of OXA-48.

We have observed quercetin, fisetin, 3′,4′,7-trihydroxyflavone, and luteolin inhibited OXA-48 carbapenemase in the ∆*acrA*, ∆*bamB*, and ∆*acrA*∆*bamB* mutant *E.coli* strains with 2–8-fold reduction in MICs, but showed no inhibitory activity against wild type *E.coli* expressing OXA-48. This is likely because of bacterial outer membrane permeability constraints. Previous studies have reported PAβN, an inhibitor of AcrAB-TolC efflux pump, increased the permeation of 3′,4′,7-trihydroxyflavone in multi-drug resistant (MDR) Gram-negative bacteria (Abdali et al., 2017; Dzotam et al., 2018). In this study, disruption of AcrAB-TolC efflux pump increased the efficacy of quercetin and 3′,4′,7-trihydroxyflavone when combined with piperacillin and imipenem, indicating that improving the bacterial outer membrane permeability resulted in the accumulation of the compound in the periplasm so that it could reach and inhibit OXA-48 effectively. Thus, improving the ability of flavonoids to cross the bacterial outer membrane is an important direction for future research.

In summary, to search for OXA-48 inhibitors, quercetin and its structural analogs were found to be potent OXA-48 inhibitors. Quercetin, fisetin, luteolin, and 3′,4′,7-trihydroxyflavone exhibited excellent inhibitory activity against OXA-48 with IC_50_ values ranging from 0.47 to 1.89 µM. Further studies revealed that quercetin inhibited OXA-48 through a reversible noncovalent inhibition mode, which was verified by enzymatic kinetics assays, ITC, and SPR assays. Structure-activity relationship studies and molecular docking suggested that the hydroxyl groups at the 3′, 4′ and 7 positions enhance hydrogen-bonding interaction with OXA-48, while the absence of a 2,3-double bond decreases the inhibitory activity. MIC tests indicated that the flavonoids restored β-lactam activity against *E. coli* strain producing OXA-48, resulting in a 2–8-fold reduction in MIC without affecting the growth of bacteria. Combination therapy of piperacillin and quercetin showed a synergy effect and could significantly decrease the bacterial load in the spleen and liver of the mice systemic infection model. The information gained here may be useful to optimize the structures of flavonoids and increase their activity in the future.

## Data Availability

The original contributions presented in the study are included in the article/Supplementary Material, further inquiries can be directed to the corresponding author.
