# Supplementary material for: Discovery of Quercetin and Its Analogs as Potent OXA-48 Beta-Lactamase Inhibitors
Source: Front Pharmacol. 2022 Jun 22;13:926104. doi: 10.3389/fphar.2022.926104 (PMC9258905; doi:10.3389/fphar.2022.926104)
Supplement: Supplementary file 1 [file DataSheet1.pdf]

Supplementary material

for

Discovery of quercetin and its analogs as potent OXA-48 beta-lactamase inhibitors

Yuejuan Zhang[a,b], Cheng Chen[c], Bin Cheng[d], Lei Gao[a,b], Chuan Qin [a,b], Lixia Zhang[e], Xu Zhang[a,b], Jun Wang[a,b], Yi Wan[a,b*]

[a] *Microbiology Institute of Shaanxi, Xi'an, Shaanxi 710043, PR China;*

[b] *Engineering Center of Qinling Mountains Natural Products, Shaanxi Academy of Sciences, Xi'an, Shaanxi 710043, PR China;*

[c] *College of Forestry, Northwest A&F University, Yangling, Shaanxi 712100, PR China;*

[d] *MOE Key Laboratory of Cell Activities and Stress Adaptations, School of Life Sciences, Lanzhou University, Lanzhou, Gansu 730000, PR China;*

[e] *Clinical Laboratory, Shaanxi Provincial People's Hospital, Xi'an, Shaanxi 710068, PR China.*

[*] *Corresponding author: Yi Wan, Phone/fax: +86-29-8235-7035, E-mail: wanyi6565@163.com.*

Table of contents

Supplementary Figure S1	The HPLC chromatograph of flavonoids	S2
Supplementary Table S1	The oligonucleotides used for cloning in the study	S5
Supplementary Figure S2	The growth curve of OXA-48-positive *E. coli* with different concentrations of piperacillin.	S6
Supplementary Figure S3	Profile of OXA-48 inhibition by flavonoids	S6
Supplementary Figure S4	Lineweaver–Burk plots of inhibition of OXA-48 by fisetin (A) and luteolin (B)	S8

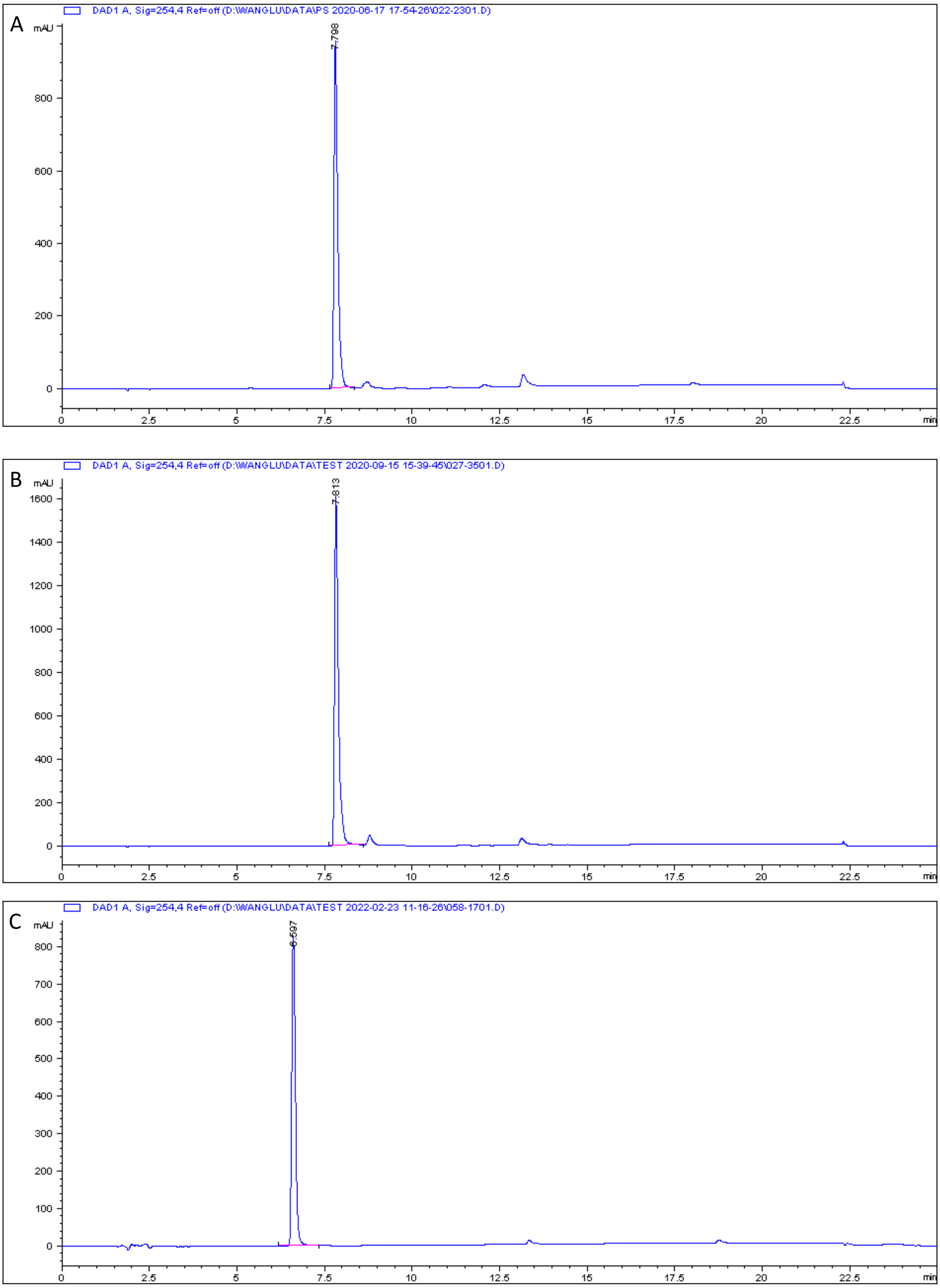
A
DAD1 A, Sig=254,4 Ref=off (D:\WANGLU\DATA\PS 2020-06-17 17-54-26\022-2301.D)
mAU
7.798
800
600
400
200
0
0
2.5
5
7.5
10
12.5
15
17.5
20
22.5
min
B
DAD1 A, Sig=254,4 Ref=off (D:\WANGLU\DATA\TEST 2020-09-15 15-39-45\027-3501.D)
mAU
7.813
1600
1400
1200
1000
800
600
400
200
0
0
2.5
5
7.5
10
12.5
15
17.5
20
22.5
min
C
DAD1 A, Sig=254,4 Ref=off (D:\WANGLU\DATA\TEST 2022-02-23 11-16-26\058-1701.D)
mAU
6.597
800
700
600
500
400
300
200
100
0
0
2.5
5
7.5
10
12.5
15
17.5
20
22.5
min

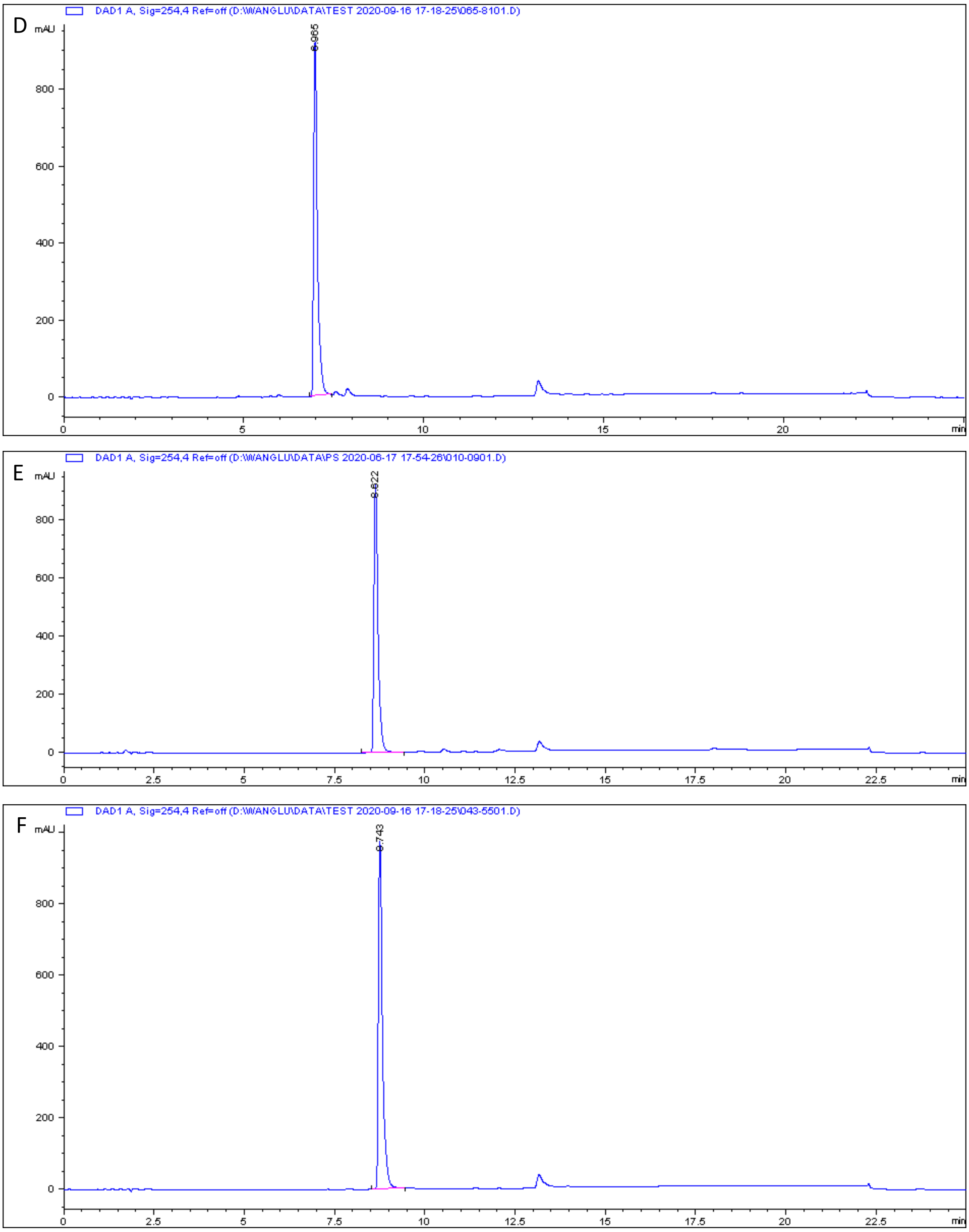
D
DAD1 A, Sig=254,4 Ref=off (D:\WANGLU\DATA\TEST 2020-09-16 17-18-25\065-8101.D)
mAU
6.965
800
600
400
200
0
0
5
10
15
20
min
E
DAD1 A, Sig=254,4 Ref=off (D:\WANGLU\DATA\PS 2020-06-17 17-54-26\010-0901.D)
mAU
8.622
800
600
400
200
0
0
2.5
5
7.5
10
12.5
15
17.5
20
22.5
min
F
DAD1 A, Sig=254,4 Ref=off (D:\WANGLU\DATA\TEST 2020-09-16 17-18-25\043-5501.D)
mAU
8.743
800
600
400
200
0
0
2.5
5
7.5
10
12.5
15
17.5
20
22.5
min

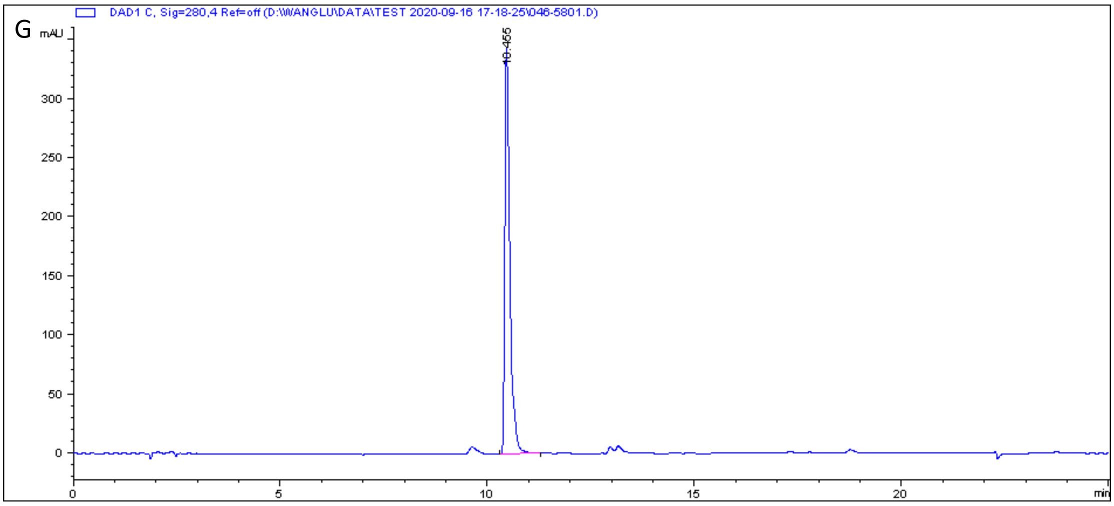
G
DAD1 C, Sig=280,4 Ref=off (D:\WANGLU\DATA\TEST 2020-09-16 17-18-25\046-5801.D)
mAU
10.455
300
250
200
150
100
50
0
0
5
10
15
20
min

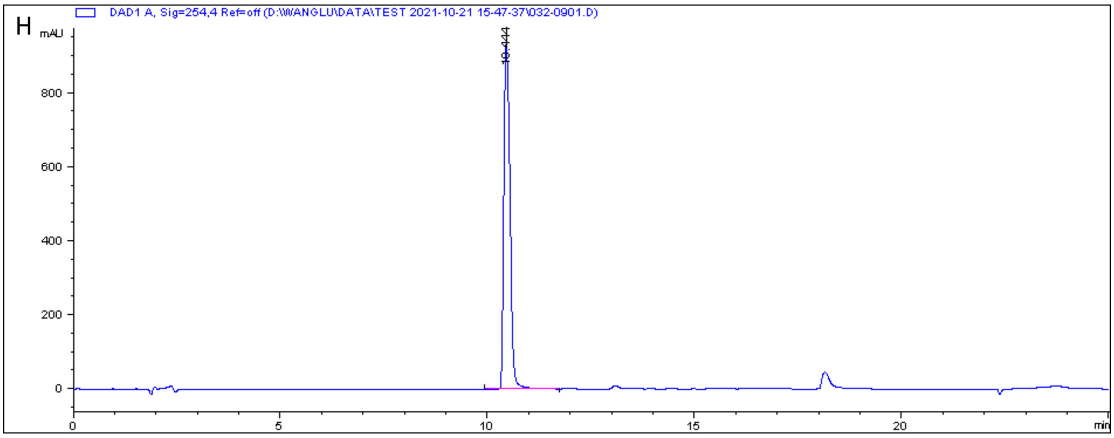
H
DAD1 A, Sig=254,4 Ref=off (D:\WANGLU\DATA\TEST 2021-10-21 15-47-37\032-0901.D)
mAU
10.444
800
600
400
200
0
0
5
10
15
20
min

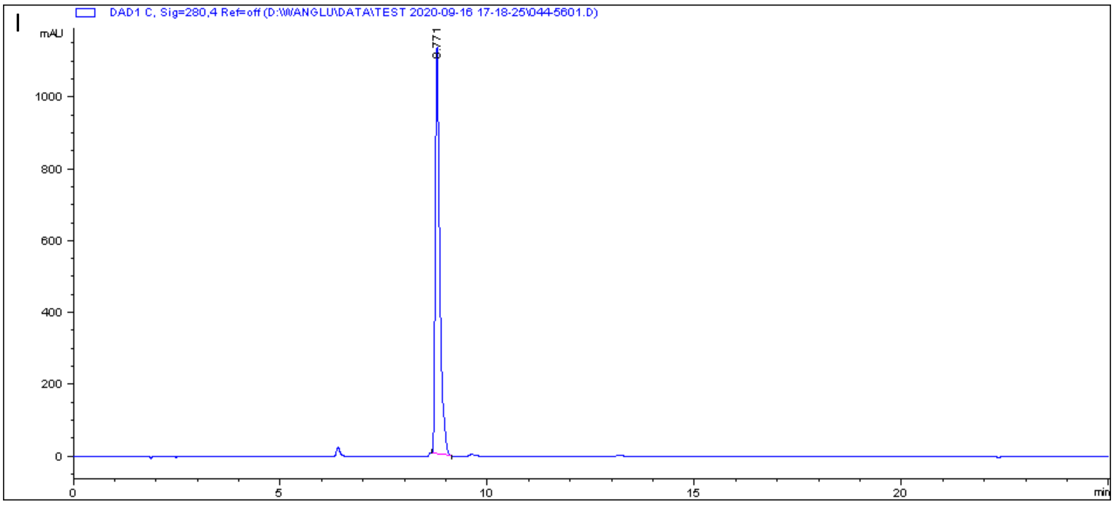
I
DAD1 C, Sig=280,4 Ref=off (D:\WANGLU\DATA\TEST 2020-09-16 17-18-25\044-5601.D)
mAU
8.771
1000
800
600
400
200
0
0
5
10
15
20
min

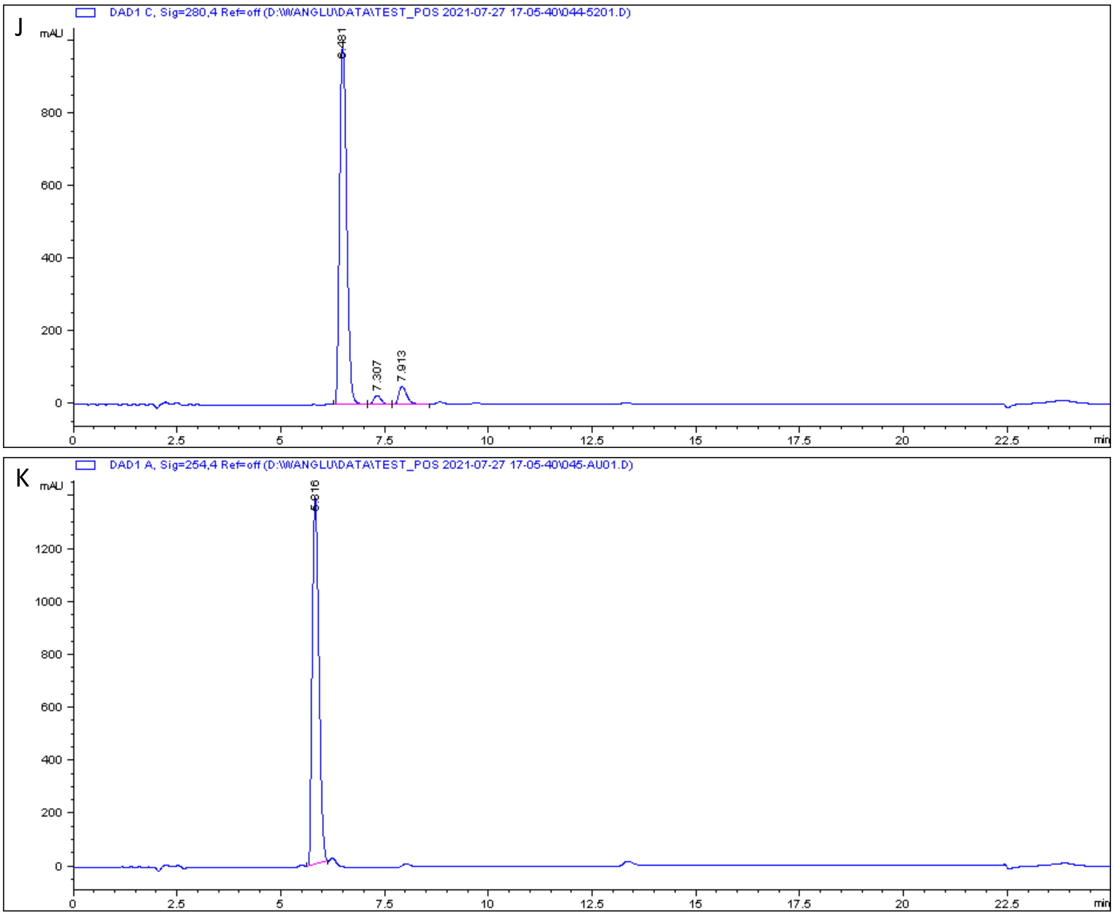

Supplementary Figure S1. The HPLC chromatograph of flavonoids. A: luteolin; B: quercetin; C: 3’,4’,7-trihydroxyflavone; D: fisetin; E: apigenin; F: kaempferol; G: Chrysin; H: galangin; I: naringenin; J: taxifolin; K: isoquercitrin.

Supplementary Table S1. The oligonucleotides used for cloning in the study.

**Primer**	**DNA sequence 5’-3’**
acrB-Fw	ATTTGTTCAGCTCATTCGGGTTCA
acrR-Rv	AGAGATTACGTTGTGCCTGTTGC
pBla-Fw	CCTTT**AGATCT**GGCCTATTGGTTAAAA AATG
pBla-Rv	CCTT**TTCTAG**AGAAGCATTTATCAGGG TTATTG

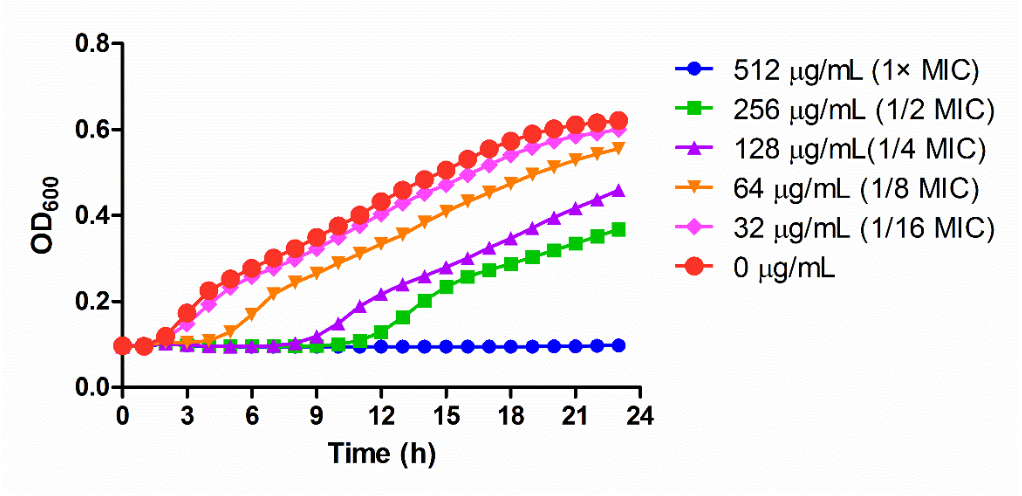

Supplementary Figure S2. The growth curve of *E. coli* BW25113 Δ*acrA*Δ*bamB* expressing OXA-48 with different concentrations of piperacillin.

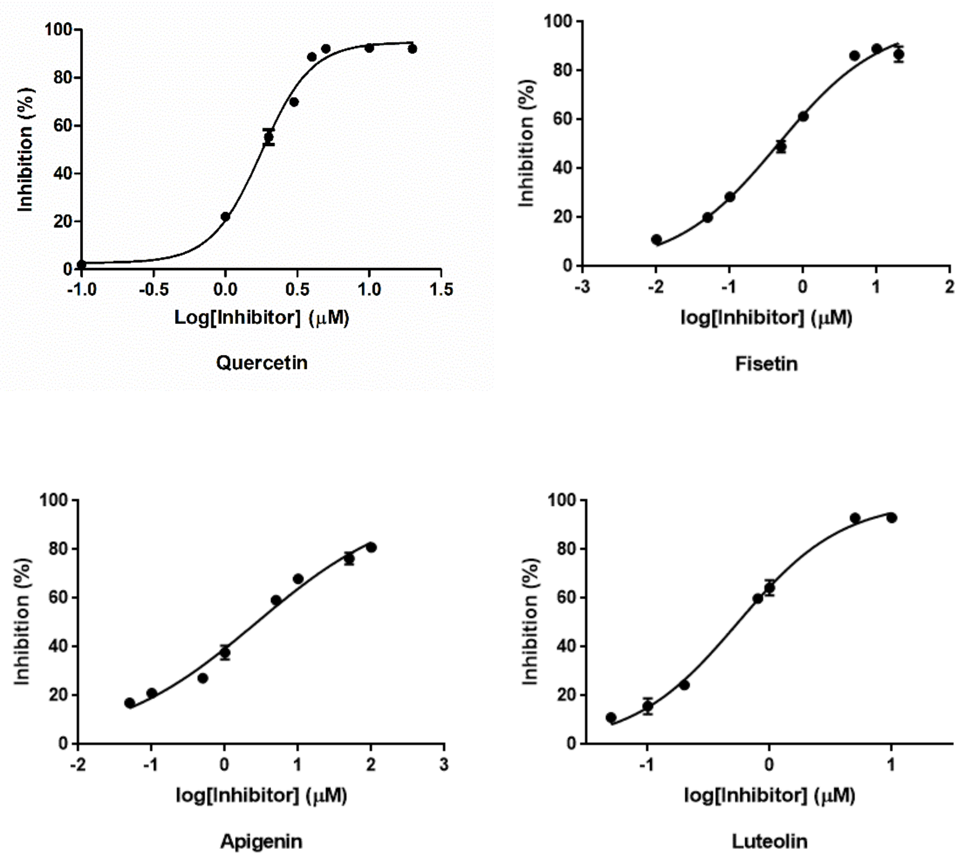

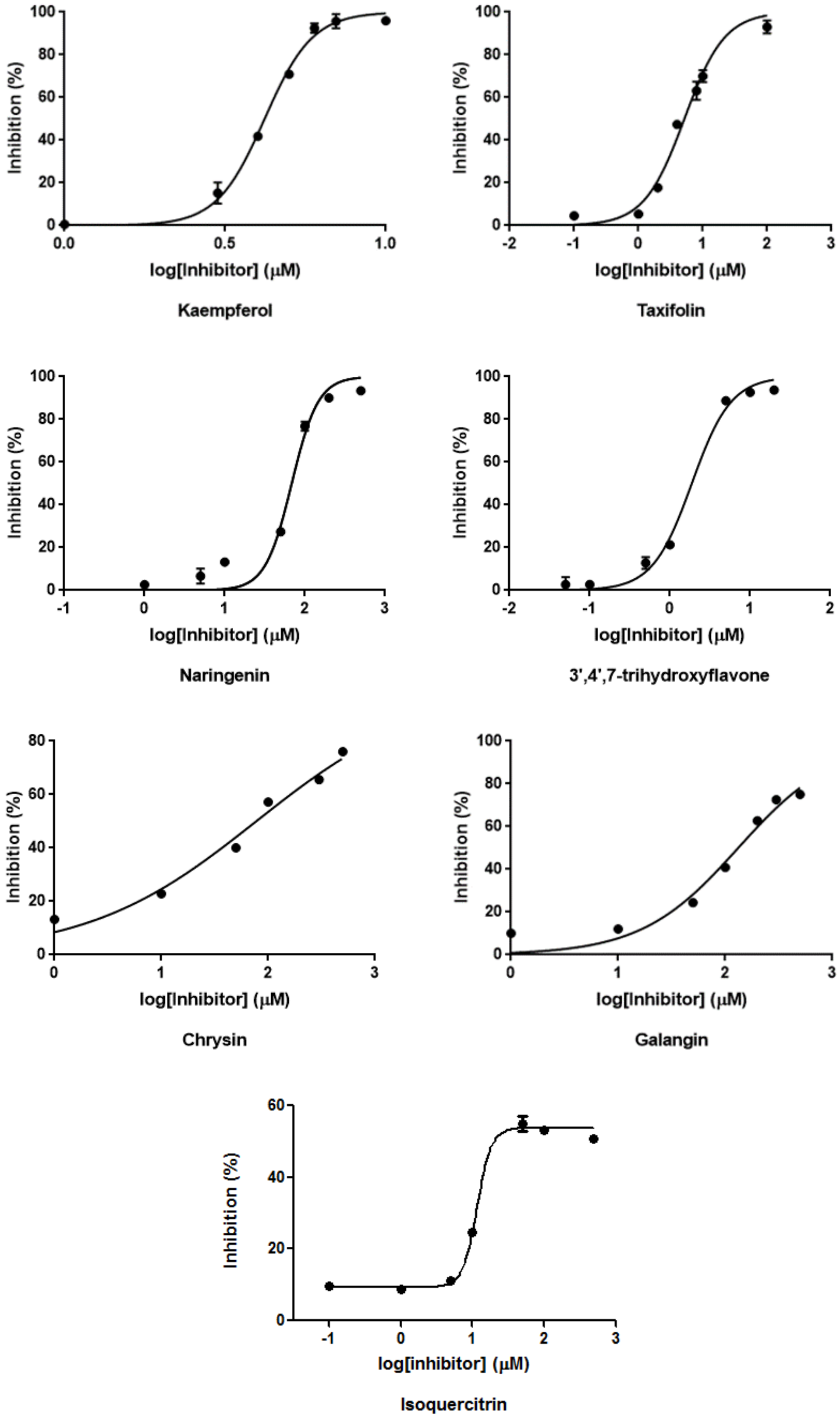

Supplementary Figure S3. Profile of OXA-48 inhibition by flavonoids.

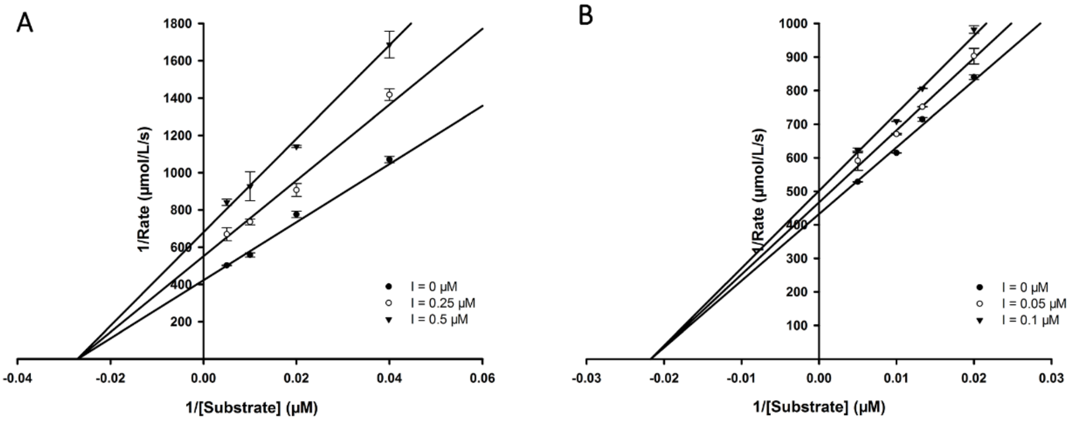

Supplementary Figure S4. Lineweaver–Burk plots of OXA-48 catalyzed hydrolysis of the substrate in the absence and presence of fisetin (A) and luteolin (B) at various concentrations.
